# Evaluating the Effectiveness of Nasoalveolar Molding in the Management of Children with Unilateral Cleft Lip and Palate: A Cohort Study

**DOI:** 10.3390/dj13090394

**Published:** 2025-08-28

**Authors:** Alba España-Guerrero, Enrique España-Guerrero, Esther Liceras-Liceras, Elena Bullejos-Martínez, Adoración Martínez-Plaza, Miguel Alaminos, Ricardo Fernández-Valadés, Antonio España-López

**Affiliations:** 1 Doctoral Program in Clinical Medicine and Public Health, University of Granada, 18071 Granada, Spain; alba@dentalos.es; 2 Department of Stomatology, Faculty of Dentistry, University of Granada, 18071 Granada, Spain; enrique@dentalos.es (E.E.-G.); ajep@ugr.es (A.E.-L.); 3 Division of Pediatric Surgery, University Hospital Virgen de las Nieves, 18014 Granada, Spain; esther.liceras.sspa@juntadeandalucia.es; 4 Craniofacial Malformations and Cleft Lip and Palate Management Unit (Unidad de Fisurados Labiopalatinos y Malformaciones Craneofaciales), University Hospital Virgen de las Nieves, 18014 Granada, Spain; adoracionmartinez@medicalpur.com; 5 University Hospital Foundation Jiménez Díaz, 28040 Madrid, Spain; ebullejos90@gmail.com; 6 Division of Oral y Maxillofacial Surgery, University Hospital Virgen de las Nieves, 18014 Granada, Spain; 7 Tissue Engineering Group, Department of Histology, School of Medicine, University of Granada, 18016 Granada, Spain; 8 Instituto de Investigación Biosanitaria ibs.GRANADA, 18012 Granada, Spain

**Keywords:** nasoalveolar molding, unilateral cleft lip and palate, nasal symmetry

## Abstract

**Background**: Children affected by unilateral cleft lip and palate (UCLP) represent a therapeutic challenge requiring the development of novel therapies, such as the implant of a bioengineered tissue—BIOCLEFT—or the use of nasoalveolar molding (NAM). The objective of this work was to evaluate the effects of NAM on the surgical and aesthetic outcomes of children with UCLP. **Methods**: A total of 36 children with UCLP treated at a craniofacial malformations management unit were evaluated, including 23 patients treated with presurgical NAM followed by palate surgical correction (NAM group) and 13 patients treated surgically without previous NAM (non-NAM group). Measurements were obtained from each patient immediately before palate surgery, including four linear measurements: nasal ala projection length (NAPL), nasal dome height (NDH), superoinferior alar groove position (S-I AGP), and nasal dome position (M-L NDP), and two angular measurements: columellar deviation (CD) and nasal bridge deviation (NBD). **Results**: When NAM was used, a significant improvement of the basilar view linear measurements of the patient’s nose was found, including the NAPL and NDH, and the frontal view linear measurement M-L NDP, but not S-I AGP. Significant improvements were also observed in the angular measurements of nasal symmetry CD and NBD. All these variables, except the S-I AGP, significantly correlated with the treatment group, and two variables—NAPL and CD—significantly contributed to generate a predictive model developed using binary logistic regression. **Conclusions**: These findings support the use of NAM to efficiently improve the nasal symmetry and the presurgical outcomes of patients with UCLP.

## 1. Introduction

With an estimated prevalence of approximately 1.5 cases per 1000 newborns, and a risk ratio of 0.989 cases per year, cleft lip and palate represent of the most common craniofacial congenital malformations in Europe [1], ranking second only to Down syndrome among congenital defects [2].

Management of children with unilateral cleft lip and palate (UCLP) is very complex, and its main objective is to restore normal morphology and function of the affected structures, particularly the nose, lip, teeth and palate, but also the structures involved in speech, breathing, and chewing, and other vital functions [3]. Most patients affected by UCLP display a severe nasal asymmetry, due to malposition of the alar base of the nose at the side of the defect [4,5,6]. This malposition is a consequence of the separation of the maxillary bone caused by incomplete fusion of the facial processes [7], which displaces muscle insertions and alters their course, ultimately leading to nasal deformity [8]. In addition, patients affected by UCLP frequently exhibit severe dental displacement and malocclusion requiring complex dental and orthodontic interventions aimed at restoring craniofacial symmetry [9].

Despite significant advances in the treatment of children with UCLP, many patients continue to experience persistent asymmetry of the nose, lip, and teeth distribution, often requiring multiple procedures that rarely achieve optimal results [10].

Several alternative treatments have been proposed to improve the results of the treatment of patients with UCLP, such as the implant of the BIOCLEFT bioengineered palate mucosa substitute [11] or nasoalveolar molding (NAM). NAM has been shown to significantly contribute to and improve the outcomes of children with UCLP, and to reduce the need for complex dental and orthodontic treatments [5,12,13,14]. This procedure is typically performed before the surgical correction of the cleft tissues, with the objective of reorienting and guiding the misaligned structures, bringing together the alveolar segments that are separated in UCLP patients [15]. Consequently, NAM is normally used as a presurgical adjunctive treatment intended to provide the surgeon with a more favorable presurgical situation [15].

In general, most reports coincide in demonstrating a positive effect of NAM applied to children with UCLP [12,16,17,18]. Several studies demonstrated that patients subjected to NAM achieve better functional and aesthetic results [19], as well as higher body mass index and weight, compared to children treated with conventional protocols that do not include NAM [20]. In addition, NAM may reduce the likelihood of requiring secondary interventions to close the maxillary gap with a bone graft [21]. However, different review studies found a lack of high-level evidence and a considerable heterogeneity across studies, suggesting the need for new research studies using highly homogeneous cohorts or patients treated with the same standardized methods and surgical procedures [22,23].

The objective of the present study was to evaluate the nasal symmetry in children with UCLP treated with or without NAM, and to determine the usefulness of this technique in achieving a more symmetric aspect of the patient’s nose.

## 2. Materials and Methods

### 2.1. Patients Included in the Study

A total of 36 children treated at the Craniofacial Malformations and Cleft Lip and Palate Management Unit of the University Hospital Virgen de las Nieves were included in this retrospective cohort study. All patients had to meet all the following inclusion criteria: (1) patients admitted at the unit between birth and 30 days of age; (2) complete UCLP; (3) absence of other syndromes or severe diseases; and (4) written informed consent signed by the parents or legal guardians. Exclusion criteria were as follows: (1) patients admitted after 30 days of age; (2) bilateral or incomplete clefts; (3) syndromic UCLP; and (4) lack of consent to participate in the study. All patients treated between 2008 and 2013 who met the inclusion criteria were enrolled in the study. The first 13 patients underwent surgical correction without previous NAM (non-NAM group), as this technique was not introduced in the Craniofacial Malformations and Cleft Lip and Palate Management Unit until 2009, whereas the last 23 patients received presurgical NAM treatment followed by surgical correction of the cleft lip and palate (NAM group). A sample size was not previously calculated for the study. Following the treatment protocols of the unit, all patients underwent surgical correction of the cleft lip between 3 and 6 months of age, while cleft palate repair was usually carried out between 15 and 18 months. All patients (NAM and non-NAM) were treated using the surgical techniques of Millard II, for lip repair, and Cutting, for nasal correction.

The study included 14 female patients (53.85% of the non-NAM group and 30.43% of the NAM group) and 22 male patients (46.15% of the non-NAM group and 69.57% of the NAM group). Mean age of the patients at the start of the treatment was 1.30 ± 0.61 days, without differences between male and female patients, whilst the age at the time of the lip correction surgery was 5.93 ± 2.26 months for females and 5.41 ± 1.36 months for males. A total of 38.46% of patients in the NAM group and 43.48% of non-NAM patients had right-side clefts, with 61.54% and 56.52% affected by left-side clefts, respectively. Regarding severity of the clefts, all patients had similar types and degrees of UCLP, with an initial cleft width of 12.62 ± 4.17 mm in both groups of patients.

For patients treated with NAM, impression molds were obtained at 1 or 2 days old. To take these impression molds, a newborn-adapted tray filled with a commercial putty silicone (Virtual Putty^®^, Ivoclar Vivadent, Schaan, Liechtenstein) was carefully applied to the patient’s face and oral cavity in order to obtain extraoral and intraoral impressions. Neither anesthesia nor sedation were required to take the impressions, due to the extensive experience of the orthodontist performing the procedure. Based on these molds, a palatal plate prosthesis was fabricated using a mixture of hard (Leocryl^®^, Leone, Florence, Italy) and soft (Eversoft^®^, Austenal, Chicago, IL, USA) orthodontic acrylic resins adapted to each patient’s morphology, with the goal of contributing to the alignment of the alveolar segments and to facilitate feeding [24]. These plates were designed by the orthodontist based on the impression casts to cover the entire palatal surface. A nasal extension was inserted into the vestibular region of the prosthesis to correct nasal deformity during the period when nasal cartilage was at its plastic phase. The NAM device was secured with adhesive tape applied to the patient’s lip and cheeks and held in place with orthodontic elastic bands. Parents were instructed on daily removal and reinsertion of the prosthesis for cleaning. The device was adjusted weekly by adding additional resin to accommodate the growth of the alveolar ridge and the movement of the nose. This process was carried out for an average of 94 days (range: 84.76–103.24 days), until the lip correction surgery was performed. All the NAM procedures were performed by the orthodontist of the unit (A.E.-L.), following standardized protocols and using identical materials. Non-NAM patients did not receive a palatal plate.

### 2.2. Obtaining Relevant Parameters of Nasal Symmetry

**Figure 1:**
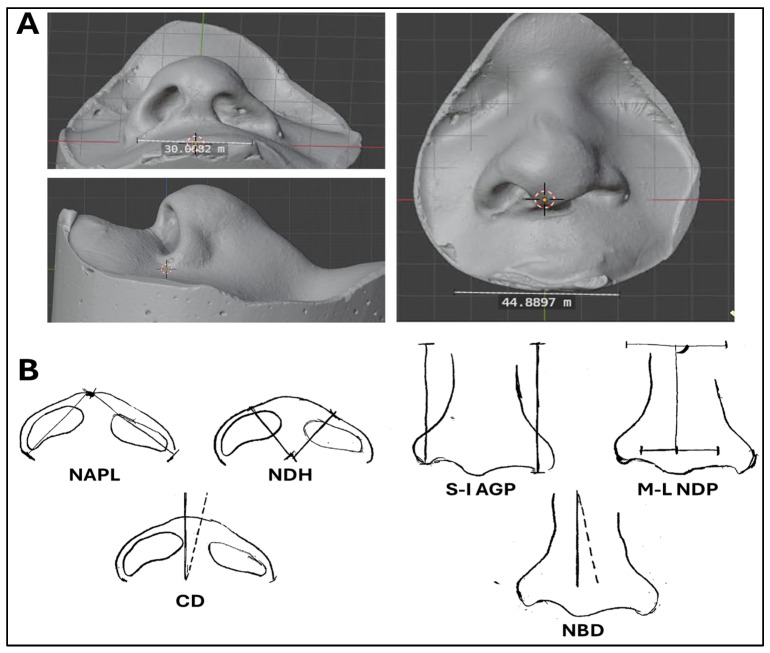
Analysis of relevant parameters measured in the present work. (**A**): Illustrative cast molds obtained from one of the patients included in the study, showing a basilar, a frontal, and a lateral view. (**B**): Parameters analyzed in the basilar and frontal views, including the NAPL (nasal ala projection length), NDH (nasal dome height), CD (columellar deviation), S-I AGP (superoinferior alar groove position), M-L NDP (mediolateral nasal dome position measurement), and NBD (nasal bridge deviation). Modified from Barillas and cols [25].

High-resolution photographs and facial cast molds were obtained from each patient approximately 1 week before the surgical correction of the palatal cleft, when patients were between 15 and 18 months of age. To do this, commercial putty silicone (Virtual Putty^®^) was applied to the patient’s face, with the child in the lateral position, covering the area from the nasal root and the inner canthi of the eyes to the upper lip. These impressions were then cast in dental stone plaster (Type IV extra-hard gypsum^®^, Proclinic, Madrid, Spain) to obtain reproductions of the patient’s nose and surrounding structures (Figure 1A).

These molds were used to assess the nasal symmetry parameters, described by Barillas and cols. Ref. [25], using a digital caliper. In order to minimize measurement error, all parameters were measured twice, and the mean value of each parameter was used in the present study. Measurements were taken directly from the plaster casts using a digital caliper, without the use of any software, and the molds were not digitized (Figure 1B).

Parameters analyzed in the cast molds were as follows:

- Basilar view linear measurements of nasal symmetry: nasal ala projection length (NAPL) and nasal dome height (NDH). The NAPL is the distance between the nasal apex and the alar grooves, measured at the cleft side and at the non-cleft side of the nose. The NDH is the distance between the base of the columella and the points defining the tip of the nose, measured at each side of the nose.
- Basilar view angular measurements of nasal symmetry: columellar deviation (CD). This parameter is quantified by determining the deviation of the columella from the true vertical—the saggital plane of the nose. This measurement was obtained by drawing a line perpendicular to the line connecting the medial canthi of the eyes, and the angle formed between this line and the actual direction of the columella was determined. The ratio of this angular deviation to 90 degrees was calculated.
- Frontal view linear measurements of nasal symmetry: superoinferior alar groove position (S-I AGP) and mediolateral nasal dome position measurement (M-L NDP). The S-I AGP was determined as the distance from an imaginary line joining both medial canthi of the eyes to the alar groove, at each side of the nose (cleft and non-cleft sides). The M-L NDP was determined at each side of the nose as the distance between each alar groove and a vertical line bisecting the intracanthal distance along the horizontal plane.
- Frontal view angular measurements of nasal symmetry: nasal bridge deviation (NBD). This parameter was assessed as the angular deviation of the nasal bridge from the true vertical determined by drawing a plane perpendicular to the line formed by joining the medial canthi. The ratio of this angular measurement to 90 degrees was found and recorded.

### 2.3. Statistical Analysis

In the first place, the dataset was analyzed to assess data reliability. An intraclass correlation coefficient (ICC) analysis was performed using Cronbach’s alpha coefficient of internal consistency, which indicated that the data were consistent and reliable (α = 0.7203). To assess intergroup comparability at baseline, chi-square tests were conducted. Results showed non-significant differences between the NAM and the non-NAM groups in terms of age, gender, or cleft side (*p* > 0.05 for all variables). In addition, a post hoc statistical power analysis of the results revealed an average value of 0.8251, indicating a good statistical power of the study.

Averages and standard deviations were calculated for each variable. For parameters measured in the cleft and the non-cleft sides of the nose (NAPL, NDH, S-I AGP, and M-L NDP), the mean absolute difference between both sides was calculated, along with the percentage of this difference relative to the total value obtained in both sides.

To compare the results of the NAM and non-NAM group, each variable was first analyzed for normality using the Shapiro–Wilk test. As we found that most parameters significantly deviated from a normal distribution, we used non-parametric statistics. Differences between the NAM vs. non-NAM groups were analyzed using the U test of Mann–Whitney. To determine the correlation of each variable with the study group (NAM or non-NAM), we used the tau correlation test of Kendall.

Then, we carried out a binary logistic regression analysis to determine whether the study group could be predicted from the measured parameters. For this purpose, only the variables that showed statistically significant differences in the Mann–Whitney U test were included in the regression model, which was constructed using the Wald backward elimination method. Variables that remained significant in the final model were used to generate a predictive equation, as previously described [26].

## 3. Results

### 3.1. Results of the Linear Measurements of Nasal Symmetry

**Figure 2:**
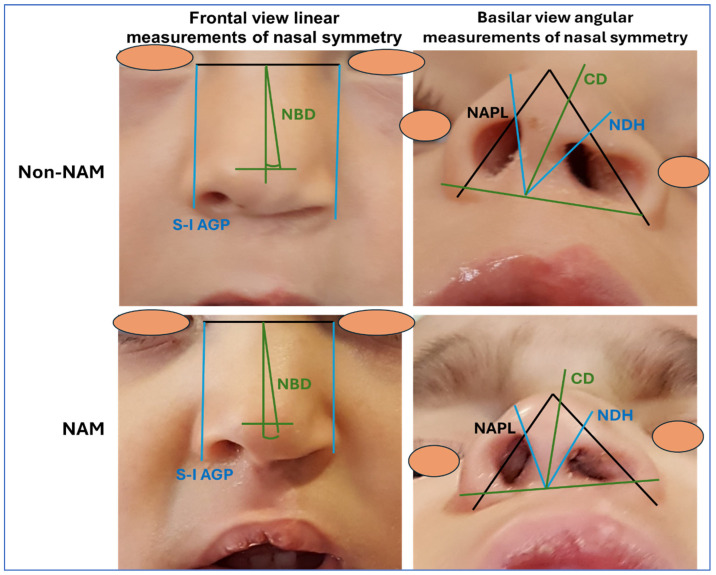
Analysis of nasal symmetry in representative images corresponding to a patient treated without nasoalveolar molding (non-NAM) and a patient subjected to nasoalveolar molding (NAM). In each case, measurements taken in the frontal and angular view are shown. NAPL: Nasal ala projection length; NDH: Nasal dome height; S-I AGP: Superoinferior alar groove position; M-L NDP: Nasal dome position; CD: Columellar deviation; and NBD: Nasal bridge deviation.

When the results of the basilar view linear measurements of the different study groups were analyzed (Table 1 and Figure 2), we found significant differences between NAM and non-NAM patients for NAPL and NDH. For NAPL, non-NAM patients showed significantly higher differences between the cleft and the non-cleft sides than NAM patients (*p* = 0.0259 for the U test of Mann–Whitney), with the cleft side tending to present larger distance than the non-cleft side. These differences remained statistically significant when male patients were analyzed separately (*p* = 0.014), but not among females (*p* > 0.05). A significant correlation was also observed between NAPL and the study group (NAM and non-NAM) (*p* = 0.0262 and r = 0.3110 for the tau test of Kendall).

Similar results were found for the NDH parameter, with non-NAM patients showing significantly higher differences between the cleft and non-cleft sides than patients subjected to NAM (*p* < 0.0001), suggesting that the NAM treatment was associated with a more symmetric nasal profile. The same behaviors were found when female and male patients were analyzed separately, with significant differences found between NAM and non-NAM patients for females (*p* = 0.0175) and males (*p* = 0.0001). Correlation between NDH and the study group was statistically significant (*p* = 0.0001 and r = 0.5571).

After the basilar view linear measurements were obtained, we analyzed the nasal symmetry using frontal view linear measurements. For the S-I AGP parameter, we found non-significant differences between NAM and non-NAM patients, either in the overall cohort of patients and when females and males were analyzed separately (*p* > 0.05). Likewise, S-I AGP values were not significantly correlated with the study group (*p* > 0.05).

In contrast, analysis of the M-L NDP parameter showed significant differences between NAM and non-NAM children. Specifically, the distance of the cleft side of the nose was significantly larger in non-NAM patients compared to those treated with NAM (*p* < 0.0001). These differences were also statistically significant when female and male patients were analyzed separately (*p* = 0.0262 for females and *p* = 0.0012 for males). A significant correlation was also found between M-L NDP and the study group (*p* = 0.0001 and r = 0.5419).

### 3.2. Results of the Angular Measurements of Nasal Symmetry

To further assess nasal deviation, nasal symmetry was analyzed using angular measurements (Table 2 and Figure 2). For the CD parameter, we found that the angular deviation of the columella was significantly higher in non-NAM children as compared to the NAM group (*p* = 0.0121), with NAM children tending to present lower deviation angles than non-NAM patients. This trend was observed in both genders, although differences were statistically significant only among males (*p* = 0.0034). Furthermore, the results obtained for CD significantly correlated with the study group (*p* = 0.0130 and r = 0.3593).

Furthermore, results of the NBD measurements revealed that non-NAM patients again demonstrated significantly greater deviation than NAM patients (*p* = 0.0216). Differences were also significant for female patients analyzed separately (*p* = 0.0175), but not for males (*p* > 0.05), and a significant correlation with the study group was found (*p* = 0.0224 and r = 0.3297).

### 3.3. Generation of a Predicting Model Using Binary Logistic Regression

Binary logistic regression was performed to evaluate the predictive value of the variables that showed statistical significance in the previous analyses. Results showed that two parameters remained significant in the final model: NAPL (*p* = 0.0204) and CD (*p* = 0.0425). Based on these variables, a predictive model was developed to estimate the likelihood of a case belonging to the NAM or non-NAM group: Likelihood of non-NAM/NAM (study group) = 1/(1 + exp − (8.151 + 1.803 × NAPL − 0.453 × CD)), where NAPL represents the nasal ala projection length thickness (difference between the cleft and the non-cleft sides) and CD represents the columellar deviation.

## 4. Discussion

The clinical implementation of the NAM therapy in the pediatric and maxillofacial surgery units has represented a significant advance in the treatment of patients with cleft lip and palate [14,27]. Presurgical NAM has demonstrated to efficiently reduce cleft width, thereby allowing the surgeon to perform the surgical repair of cleft structures with a higher degree of success by reducing presurgical deformities [12,27,28]. In general, the efficiency of the NAM procedure relies on the high plasticity and proliferation rate of the maxillofacial structures of the newborns [10].

Although the clinical effectiveness of the NAM has been confirmed by several clinical studies and some metanalyses [12,16,17,18], most published works are highly heterogeneous regarding the methods used both for the NAM procedure and for the surgical repair of the defect, highlighting the need for further standardized studies [13,29]. Moreover, some previous studies reported insufficient data, which limits the possibility of conducting proper quantitative analysis of soft tissue structures [22,23].

In the present work, we performed an analysis of children with UCLP treated in the same pediatric surgery unit, all of whom underwent the same surgical procedure performed by the same pediatric surgeon. Furthermore, all patients were treated by the same orthodontist, and all NAM patients were treated by the same standardized NAM procedure. The high level of homogeneity within the cohort of patients described in this work could contribute to establishing the usefulness of the NAM technique without the heterogeneity associated with some of the previously published papers [13].

Overall, results of our study confirmed that the NAM technique could contribute to improving the clinical results of children affected by UCLP, as previously reported [5,25,30]. When the basilar view lineal parameters of the patients’ nose were analyzed, we found that NAM significantly improved both NAPL and NDH, and a correlation between both measurements and the use of NAM was found. These parameters reflect nasal symmetry in terms of the length and high of the nasal ala on the cleft side, and obtaining similar values of NAPL and NDH on both sides is essential for an optimal aesthetic result [31]. It is well known that the nasal ala of children with cleft lip and palate is typically enlarged at the cleft side of the face, with inferior and lateral displacement, due to depression and misalignment of the lateral alar cartilage [4,5]. In our study, NAM treatment successfully remodeled the alveolar process and displaced it towards the midline. This may explain why the NAPL and NDH parameters became more similar between the cleft and the non-cleft sides, as the nasal ala on the cleft side, which is anatomically linked to the alveolar process, is also expected to become closer to the midline. Interestingly, the same beneficial effects of NAM were found in male patient, and, in the case of the NDH, also in females. These results are in agreement with previous reports demonstrating the beneficial effect of the NAM treatment on both the NAPL and NDH basilar view lineal parameters [25], and are aligned with studies showing a reduction in alar width and cleft gap in patients treated with NAM [32]. Furthermore, the ability of NAPL to efficiently predict NAM use in our binary logistic regression model reinforces the strong association of this parameter with the NAM treatment.

Along with the basilar view lineal parameters, we also evaluated two relevant frontal view nasal measurements, including the S-I AGP and M-L NDP. Like NAPL and NDH, the S-I AGP and M-L NDP are directly related to the position of the lower lateral cartilage, which is attached to the caudal region of the upper lateral cartilage [4]. In patients with UCLP, displacement of these cartilages typically results in elongation of the S-I AGP and M-L NDP on the cleft side of the nose [5]. In our study, we found that M-L NDP was significantly improved in NAM patients as compared to non-NAM patients, and the length of the cleft side tended to approximate that of the non-cleft side, resulting in better nasal symmetry. These results were confirmed in both female and male patients. However, we found that the NAM procedure was not correlated with the S-I AGP parameter, with few differences between NAM and non-NAM patients. These findings suggest that NAM might contribute to the effective displacement of the alar cartilage of the cleft side towards the midline, but its impact on vertical displacement appears to be very limited. In general, the results found for M-L NDP are in agreement with previous reports suggesting the beneficial effect of NAM in displacing the cleft side nasal ala to a more medial position [25]. However, our results regarding the lack of improvement in S-I AGP contrast with some previous studies. One possible explanation is the high variability observed in our cohort, with considerable standard deviation of the S-I AGP percentages, which may have masked potential effects, highlighting the need for future studies using larger groups of patients.

Furthermore, nasal symmetry was also characterized by measuring two parameters associated with the angular displacement of the nose, such as the CD and NBD. These parameters are particularly relevant, as patients with UCLP typically present a deviation of the caudal septum of the nose towards the non-cleft side, associated with a shortening of the columella on the cleft side of the nose [33]. For both the CD and NBD, we found that the NAM treatment was significantly associated with a reduction in the angular deviations typically found in patients affected by UCLP, with CD emerging as a predictor of group allocation in the regression model. As for the previous parameters, these results are partially consistent with previous studies published by Barilla and cols., in which the NAM treatment was significantly associated with an improvement in NBD, but not in CD. Another interesting finding of our study is the presence of gender-related differences, with CD significantly associated with NAM treatment only in males, and NBD only in females. Again, we might hypothesize that these differences could be related to the limited sample size in each group of patients when stratification by gender is performed.

In general, our study supports the use of NAM in children born with UCLP, showing a significant association between NAM and most of the nasal symmetry parameters analyzed in the present work. Moreover, the study allowed us to efficiently predict the patient’s group (NAM or non-NAM) based on two key parameters—NAPL and CD—thus reinforcing the association of these parameters with NAM treatment. These findings suggest that the use of NAM may improve the presurgical situation of patients with UCLP, and it is likely that this improvement could facilitate the surgical procedure and contribute to more favorable long-term outcomes. Nevertheless, it is still unknown whether combining NAM with novel regenerative approaches, such as the implant of a BIOCLEFT bioartificial human palate mucosa substitute [11], could lead to optimal final results after craniofacial growth and development are complete.

One of the main strengths of this study is the homogeneity of the treatment applied to all patients included, in contrast with some previous studies [13], as well as the broad number of parameters determined in each patient. However, the study also has some limitations, including the relatively small sample size, particularly when patients were stratified by gender. In addition, longer follow-up studies are needed to evaluate the utility of NAM in craniofacial development over time and its potential effects on non-nasal structures such as the lip. Future research should aim to confirm these findings and to explore the possibility of combining NAM with regenerative approaches, such as the implantation of BIOCLEFT bioengineered tissue, to improve the final outcomes of children with UCLP.

## 5. Conclusions

Our study suggests that NAM could contribute to improving nasal symmetry in UCLP patients treated with this procedure, with a significant correlation found with most parameters analyzed, including NAPL, NDH, M-L NDP, CD, and NBD, although its effect on S-I AGP was not significant.

## Figures and Tables

**Table 1:** Absolute values and differences obtained for the linear measurements of nasal symmetry analyzed in this work. The *p* value for the statistical comparison of the percentage of differences in the cleft side vs. the non-cleft side of each patient is shown in the lower row. NAPL: Nasal ala projection length; NDH: Nasal dome height; S-I AGP: Superoinferior alar groove position; and M-L NDP: Nasal dome position. Statistically significant differences are highlighted with asterisks (*).

		NAPL			NDH			S-I AGP			M-L NDP		
		All	Females	Males	All	Females	Males	All	Females	Males	All	Females	Males
Non-NAM	Distance Cleft Side	17.85 ± 3.58	16.75 ± 3.77	19.13 ± 3.16	9.72 ± 2.69	9.22 ± 3.27	10.31 ± 1.94	25.92 ± 4.89	24.79 ± 5.54	27.24 ± 4.08	7.86 ± 1.66	8.14 ± 1.46	7.54 ± 1.96
	Distance Non-Cleft Side	16.39 ± 4.24	16.4 ± 4.93	16.38 ± 3.74	13.96 ± 2.28	13.58 ± 2.04	14.41 ± 2.65	27.93 ± 4.22	27.87 ± 4.78	28 ± 3.93	5.06 ± 1.93	5.45 ± 2.56	4.6 ± 0.76
	Mean Difference Cleft Side vs. Non-Cleft Side	1.46 ± 3.85	0.35 ± 4.67	2.76 ± 2.4	−4.24 ± 2.47	−4.36 ± 2.37	−4.1 ± 2.8	−2.01 ± 3.28	−3.07 ± 3.21	−0.76 ± 3.17	2.81 ± 2.7	2.69 ± 3.62	2.94 ± 1.31
	% Difference Cleft vs. Non-Cleft Side	5.05 ± 11.35	2.09 ± 13.51	8.49 ± 7.99	−18.91 ± 11.75	−21.05 ± 13.24	−16.41 ± 10.34	10.45 ± 52.52	−10.06 ± 56.8	34.38 ± 38.39	23.33 ± 44.55	5.98 ± 45.71	43.57 ± 36.64
NAM	Distance Cleft Side	18.89 ± 3.26	18.19 ± 3.62	19.2 ± 3.16	14.29 ± 2.84	13.95 ± 2.51	14.44 ± 3.04	26.63 ± 3.94	27.69 ± 3.71	26.16 ± 4.07	6.82 ± 1.6	6.93 ± 2.01	6.77 ± 1.47
	Distance Non-Cleft Side	18.74 ± 3.12	17.59 ± 3.09	19.25 ± 3.09	15.13 ± 3.14	14.84 ± 3.03	15.26 ± 3.28	26.97 ± 3.82	27.46 ± 3.67	26.75 ± 3.98	6.45 ± 1.57	6.81 ± 2	6.3 ± 1.39
	Mean Difference Cleft Side-Non-Cleft Side	0.15 ± 1.19	0.6 ± 1.44	−0.05 ± 1.05	−0.84 ± 1.5	−0.89 ± 1.3	−0.82 ± 1.61	−0.34 ± 1.64	0.24 ± 1.26	−0.59 ± 1.75	0.36 ± 1.11	0.12 ± 0.97	0.47 ± 1.18
	% Difference Cleft vs. Non-Cleft Side	0.33 ± 3.12	1.47 ± 3.46	−0.17 ± 2.94	−2.81 ± 4.75	−2.9 ± 4.71	−2.77 ± 4.93	6.27 ± 51.05	21.91 ± 47.99	−0.58 ± 52.31	7.83 ± 50.69	21.48 ± 50.27	1.86 ± 51.31
Non-NAM vs. NAM	*p* Value (% Difference)	0.0259 *	0.5350	0.0104 *	<0.0001 *	0.0175 *	0.0001 *	0.5804	0.9015	0.2030	<0.0001 *	0.0262 *	0.0012 *

**Table 2:** Absolute values for the angular measurements of nasal symmetry analyzed in this work. The *p* value for the statistical comparison of the cleft side vs. the non-cleft side of each patient is shown in the lower row. CD: Columellar deviation; and NBD: Nasal bridge deviation. Statistically significant differences are highlighted with asterisks (*).

	CD			NBD		
	All	Females	Males	All	Females	Males
Non-NAM	9.62 ± 3.75	8.57 ± 4.86	10.83 ± 1.47	7.38 ± 3.38	8.71 ± 1.89	5.83 ± 4.22
NAM	6.39 ± 3.39	6.43 ± 3.82	6.38 ± 3.32	4.61 ± 2.74	4.71 ± 2.75	4.56 ± 2.83
Non-NAM vs. NAM	0.0121 *	0.3829	0.0034 *	0.0216 *	0.0175 *	0.6931

## Data Availability

Data supporting reported results can be found in the European repository Zenodo at https://doi.org/10.5281/zenodo.16495220.

## Abbreviations

The following abbreviations are used in this manuscript:

CD	Columellar deviation
M-L NDP	Nasal dome position
NAM	Nasoalveolar molding
NAPL	Nasal ala projection length
NBD	Nasal bridge deviation
NDH	Nasal dome height
S-I AGP	Superoinferior alar groove position
UCLP	Unilateral cleft lip and palate
